# Endometriosis: benign, malignant, or something in between?

**DOI:** 10.18632/oncotarget.21051

**Published:** 2017-09-19

**Authors:** M. Herman Chui, Tian-Li Wang, Ie-Ming Shih

**Affiliations:** Ie-Ming Shih: Departments of Pathology, Gynecology and Obstetrics, Johns Hopkins Medical Institutions, Baltimore, MD, USA

**Keywords:** KRAS, ARID1A

The transformation of a normal cell into a cancer cell is due to the progressive acquisition of driver mutations and epigenetic alterations, accompanied by changes in cellular morphology and tissue architecture. These morphologic alterations, recognized since the 19^th^ century by Virchow and other pathologists, form the basis for routine histologic examination of tissues in clinical practice, providing the means to diagnose and sub-classify neoplastic lesions. Recent work leveraging technologic advancements in next-generation sequencing and ultra-sensitive approaches for rare mutation detection has challenged our pre-conceived notions of the separation between benign and malignant disease.

In a recently published article from the *New England Journal of Medicine* [1], investigators identified somatic cancer-associated mutations, including recurrent mutations in *KRAS* and *ARID1A* in cases of deep-infiltrating endometriosis (DIE) without concurrent malignancy, as well as isolated cases harbouring *PIK3CA* and *PPP2R1A* mutations. Endometriosis is a common disease of women, causing pelvic pain and infertility. It is characterized by the presence of endometrial tissue, composed of epithelial glands and endometrial stromal cells, outside the uterus. The pelvic peritoneum and pelvic organs (including ovaries, fallopian tubes, and uterine ligaments) are the most commonly involved sites. DIE refers to cases where ectopic endometrium is found beneath the peritoneal layer, involving deep soft tissues or organs, such as the muscular layers of the bowel wall or urinary bladder. The current prevailing hypothesis posits that endometriosis results from retrograde menstruation, causing endometrial tissues to deposit and survive outside the uterus.

Despite its name (not to mention the ominous acronym), the infiltrative pattern of DIE does not resemble the destructive invasion of cancer cells, which is typically associated with a stromal desmoplastic reaction, within which are cytologically atypical tumor cells adopting an angulated configuration. In endometriosis, the tissue is morphologically normal, but misplaced. This puzzling discordance between genotype and morphologic phenotype recalls similar observations of *KRAS* mutations in normal colonic mucosa [2] and *TP53* mutations in normal fallopian tube epithelial cells [3], which are thought to represent the earliest pre-malignant genetic lesions at risk of malignant transformation. Paradoxically, DIE rarely, if ever, leads to cancer.

In contrast, there is a well-established association between ovarian endometriosis (or endometrioma) and the development of ovarian endometrioid and clear cell carcinomas [4]. *KRAS*, *ARID1A*, and *PIK3CA* are also commonly mutated in these tumors. The “perfect soil” of the ovarian microenvironment likely facilitates the transformation of endometriosis into endometriosis-related neoplasms.

What then is the role of cancer driver gene mutations in the pathogenesis of endometriosis? For mutated *KRAS*, it does not appear to act as an oncogene in this context, as its effects are non-tumorigenic and do not affect proliferation. Nor does KRAS confer self-sufficiency to growth signals; like eutopic endometrium, endometriotic lesions retain sensitivity to hormones, and anti-estrogen treatment causes their regression, as demonstrated in a *kras*-driven mouse model [5], and is an available therapeutic option in the clinic. One potential explanation may be that cell-intrinsic mechanisms suppress oncogenic activation of the Ras-MAPK pathway, thereby avoiding “oncogene-induced senescence”. Instead, the anti-apoptotic function of KRAS signalling becomes predominant [6], which is essential for preventing anoikis of endometrial cells in transit from their native environment to extrauterine sites. Similar speculations could be made for the other mutated genes, and in all cases, further mechanistic studies will be needed to confirm or refute these hypotheses.

A particularly novel finding from this study is that mutations in *KRAS* were found exclusively in endometrial glands and not in the associated stromal cells, suggesting that the glandular and stromal cells are unlikely to be clonally related [1]. The theory that endometriosis originates from a bipotent stem/progenitor cell capable of glandular and stromal differentiation, however, cannot be completely discounted; in this scenario, there would have to be a selective pressure for the epithelial progeny to acquire the driver mutation soon after the first asymmetric division leading to lineage divergence. More likely, in endometriosis, stromal cells that support survival of (mutant) endometrial glands are derived from eutopic endometrium or through differentiation of blood-borne mesenchymal stem cells, analogous to the ‘seed-and-soil’ hypothesis of cancer metastasis.

Interestingly, droplet digital PCR identified identical *KRAS*^G12D^ mutations in three anatomically distinct endometriotic lesions in one patient. Two possible explanations can account for this observation. First, since there are only a handful of hotspot mutations causing activation of KRAS, it is possible that the mutations arose independently of each other, and coincidentally happened to be identical. The more parsimonious, and in our opinion more likely, explanation is that the lesions are clonally related. The latter explanation raises the possibility that normal-appearing but *KRAS*-mutant endometrial cells in the uterus are prone to dissemination or “metastasis”, as demonstrated previously in a *kras*-mutant mouse model [7]. As *KRAS* mutations were not detected in the normal endometrium in this patient, the implication is that cells from a single endometriotic lesion may migrate and establish other lesions carrying the identical mutation. It is also conceivable that all lesions originated from normal endometrium, but the mutated cells were so exceedingly rare that they were not detected using current methodology. More thorough tissue sampling and higher depth of sequencing, preferably from fresh tissues, will be necessary for clarification of this issue.

The use of orthogonal methods (including targeted sequencing, digital droplet PCR assays and the Safe-Sequencing System) for validation of exomic sequencing is a notable strength of the study, which represents the amalgamation of data from separate cohorts collected and analyzed independently. Laser capture microdissection to separate epithelial and stromal components followed by determination of allelic frequency by digital droplet PCR were instrumental for one of the major insights gained from the study – that mutations were confined to the epithelial glands. The major limitation of this work, being a proof-of-concept study, relates to the sample size; additional sequencing of larger cohorts will be necessary to estimate mutation frequencies and to perform correlative analyses with clinical features.

In summary, as with all paradigm-shifting discoveries, the study raises more questions than answers. Should endometriosis be considered a ‘benign’ neoplasm, which harbors oncogenic driver mutations, along with the capacity for invasion and potentially for distant metastasis? Although exhibiting classic hallmarks of cancer, it is not lethal, is morphologically normal, and does not form an expansile tumor mass. The recent findings invite us to revisit our notions of what constitutes cancer, and should re-ignite interest in the biology of endometriosis, an entity which could aptly be described as “a riddle, wrapped in a mystery, inside an enigma.” But perhaps there is a key (to continue paraphrasing Sir Winston Churchill). The key is to elucidate the functional role of these cancer driver mutations in endometriosis and to correlate genotype with clinical outcomes, including response to treatment.
